# Susceptibility evaluation and PK/PD integration of tulathromycin against *Actinobacillus pleuropneumoniae* during the mutant selection window

**DOI:** 10.3389/fvets.2024.1407907

**Published:** 2024-07-03

**Authors:** Hongjuan Wang, Longfei Zhang

**Affiliations:** ^1^College of Animal Science and Veterinary Medicine, Henan Institute of Science and Technology, Xinxiang, China; ^2^Institute of Traditional Chinese Veterinary Medicine, College of Veterinary Medicine, Gansu Agricultural University, Lanzhou, China

**Keywords:** tulathromycin, *Actinobacillus pleuropneumoniae*, pharmacokinetics/pharmacodynamics, MSW, MPC

## Abstract

**Introduction:**

*Actinobacillus pleuropneumoniae* (APP) is a serious pathogen that affects the development of livestock breeding. Due to excessive use of antimicrobial drugs, many multidrug-resistant bacteria have emerged and spread, which have threatened the livestock industry. Therefore, we established a peristaltic pump infection model (PPIM) to evaluate the susceptibility change and pharmacokinetic/pharmacodynamic (PK/PD) integration of tulathromycin against APP during the mutant selection window (MSW) for preventing the emergence of mutant-resistant bacteria.

**Methods:**

The 99% minimum inhibitory concentration (MIC_99_) and mutant prevention concentration (MPC) of tulathromycin against APP were measured using the agar-plate method. After the model of dynamic infection had been established based on tulathromycin data in lungs, different dosages were administered to make the drug concentrations located in different parts of the MSW. The population and sensitivity of APP were monitored. Tulathromycin concentrations were measured by high-performance liquid chromatography-tandem mass spectrometry. Finally, a sigmoid E_max_ model was used to analyze the relationships between PK/PD parameters and antibacterial effects.

**Results and discussion:**

The values of MIC, MIC_99_, and MPC of tulathromycin against APP were 2, 1.4, and 44.8 μg/mL, respectively. The PPIM was stable. An elimination effect without regrowth was observed at 5.6 to 44.8 μg/mL (−4.48 to −7.05 Log_10_ CFU/mL, respectively). The MIC of APP increased 32-fold at 8 MIC_99_. AUC_168 h_/MIC_99_ had the best fit with the antibacterial effect (*R*^2^ = 0.9867). The AUC_168 h_/MIC_99_ required to achieve bacteriostatic, bactericidal, and clearance effects were 1.80, 87.42, and 198 h, respectively. Our results could provide guidance for the clinical application of tulathromycin to treat APP infection and avoid the generation of drug-resistant bacteria.

## 1 Introduction

*Actinobacillus pleuropneumoniae* (APP) can cause porcine contagious pleuropneumonia (PCP). The latter can result in the mass death of pigs worldwide, which would seriously affect the pig-production industry and cause huge economic losses (1–4).

Vaccination is an effective strategy for preventing PCP. However, APP has many serotypes, and cross-protection is poor (5, 6). Therefore, antimicrobial drugs (e.g., cephalosporins, fluoroquinolones, and macrolides) are commonly used to treat PCP.

However, excessive application of antimicrobial agents has led to the emergence of multidrug resistant (MDR) bacteria (7, 8). The common solutions to address MDR include the development of new drugs, reformation of susceptibility breakpoints, drug combinations, and optimization of dosage regimens. However, the time required to develop new drugs cannot keep pace with the speed at which bacteria develop resistance. Therefore, it is a more practical approach to preventing the generation of resistant bacteria by optimizing the dosage regimen based on the integration between the pharmacokinetic/pharmacodynamic (PK/PD) parameters and antibacterial effect (9–12).

The mutant selection window (MSW) represents a range of drug concentrations between the minimum inhibitory concentration (MIC) and mutant prevention concentration (MPC). Analyses of the relationships between MIC-based or MPC-based PK/PD parameters and changes in bacterial sensitivity are important for inhibiting the generation and spread of drug-resistant bacteria (13–16).

Tulathromycin is a macrolide drug approved for the treatment of respiratory diseases in pigs. The PK/PD of tulathromycin against APP has been analyzed in tissue-cage fluid from piglets (17). However, the PK characteristics of tulathromycin in tissue-cage fluid from pigs are obviously different from those in lung tissue (target tissue of APP infection). In addition, establishing a lung-infection model in pigs is difficult. The use of a peristaltic pump to simulate the PK of target tissue could be an important solution for PK/PD integration (14, 18). Moreover, in our previous study, we found that the MSW of tulathromycin against APP was wider (1.4, and 44.8 μg/mL) compared with cephalosporins and fluoroquinolones which more easily result drug resistance mutations. Therefore, it is very necessary to carry out drug resistance prevention study of tulathromycin against APP.

Therefore, in this study, we aimed to establish a peristaltic pump infection model (PPIM) according to the PK of tulathromycin in pig lungs. We wished to analyze the changes in bacterial sensitivity within and outside the MSW as well as the antibacterial effect of tulathromycin against APP. Our results could guide the formulation of dosage regimens to prevent the emergence and spread of resistant pathogens.

## 2 Materials and methods

### 2.1 Bacteria, reagents and equipment

The standard strain of APP CVCC259 and *Staphylococcus aureus* ATCC29213 were purchased from the China Veterinary Culture Collection Center (Qingdao, China). Tulathromycin powder (99.8%) was provided by Shandong Lukang Shelile Pharmaceuticals (Shandong, China). Tryptic soy broth (TSB) and Mueller–Hinton agar (MHA) were provided by Guangdong Huankai Microbiology Technology (Guangdong, China). Nicotinamide adenine dinucleotide (NAD) was sourced from Beijing Puboxin Biotechnology (Beijing, China). Fetal bovine serum (FBS) was provided by Guangzhou Ruite (Guangzhou, China). A peristaltic pump (BT100-1F), pump head (DG-2-B/D, 10 rollers), ratchet card, and rubber hose (inner diameter ≤ 3.17 mm, wall thickness = 0.8–1 mm) were purchased from Baoding Lange Constant Current Pump (Baoding, China). A fiber dialysis tube (Float A-Lyzer, 1000 KD, 10 mL) was purchased from MilliporeSigma (Burlington, MA, USA).

### 2.2 Detection of MIC, MIC_99_, and MPC

APP was cultured in TSB and MHA supplemented with 4% FBS and 1% NAD and placed in an incubator or shaker (180–200 rpm) at 37°C in an atmosphere of 5% CO_2_.

For the counting of APP populations, the original bacterial solution was serially diluted (10-fold) from 10^−1^ to 10^−6^. Then, 20 μL of each dilution was inoculated into MHA and cultured for 18–20 h. The number of bacteria (colony-forming unit (CFU)/mL) was determined with a limit of detection of 50 CFU/mL.

For MIC determination, logarithmic-phase bacterial suspensions containing APP at 10^6^ CFU/mL were inoculated into drug-containing MHA (2-fold dilution from 0.125 to 16 μg/mL) and cultured for 18–20 h. The minimum drug concentration without bacterial growth was defined as the MIC.

For MIC_99_ determination, drug containing MHA was prepared at a MIC from 90 to 50% (10% linear dilution). Logarithmic-phase APP was serially diluted (10-fold) from 10^−6^ to 10^−1^ CFU/mL, inoculated into blank and drug-containing MHA, and cultured for 18–20 h. The MIC_99_ was calculated as the concentration that inhibited the growth of bacteria by 99%.

For MPC determination, drug containing MHA at 1–16 MIC was prepared. Logarithmic-phase APP (100 mL) were enriched by centrifugation (5,000 × *g*, 20 min, 4°C) and resuspended in 1 mL of TSB to make the final suspension of 1.5 × 10^11^ CFU/mL. The bacterial suspension (100 μL) was inoculated into drug-containing MHA and cultured for 72 h. The minimum drug concentration without bacterial growth was defined as the MPC_pr_. Then, drug-containing MHA was prepared from 1 to 50% MPC_pr_ by a 10% linear decrease, and tested as the MPC_pr_ method to determine the MPC.

### 2.3 PPIM establishment

The PPIM was established according to our previous method (18). Briefly, the central chamber was a three-neck bottle and placed in a beaker containing water on a constant-temperature magnetic stirrer (100 rpm, 37°C). The two side-arms were connected by peristaltic pumps and rubber tubes to a storage chamber (fresh TSB culture medium) and an elimination chamber (for collection of waste liquid), respectively. The inner part of the middle arm was connected to a dialysis tube containing bacterial solution (10 mL) and used to administer drugs and collect samples for measurement of the drug concentration and APP population. The flow rate (Q) was set according to the following formula:

$$Q=K_{el}\times Vc\;\mathrm{and}\;K_{el}=0.693/t_{1/2\beta }$$

where K_el_ is the elimination rate constant, t_1/2β_ is the terminal half-life, and Vc is the volume of the TSB and dialysis tube in the central chamber. We defined t_1/2β_ as 142 h according to the value reported by Benchaoui et al. (19). After the flow rate had been set, the device was run for 2 h to stabilize. Then, logarithmic-phase APP (10^8^ CFU/mL) were added to the dialysis tube.

### 2.4 MIC of APP and measurement of drug concentration

According to MIC_99_ and MPC, eight dosing groups (0, 0.7, 1.4, 2.8, 5.6, 11.2, 22.4, and 44.8 μg/mL) were applied. We needed to balance the drug concentration in the dialysis tube rapidly. Hence, at the beginning of the test, an identical drug dose was added to the central chamber and dialysis chamber simultaneously. Then, 0.1 mL of the bacterial suspension from the dialysis chamber and 1 mL from the central chamber were collected at 0, 1, 3, 6, 9, 12, 24, 36, 48, 72, 96, 120, 144, and 168 h. The same volume of blank TSB was added to make the final volume identical. The number of APP were counted by the agar-plate method. The drug concentration was measured using high-performance liquid chromatography–tandem mass spectrometry (HPLC-MS/MS). Then, time–kill curves and drug concentration–time curves were created.

To monitor changes in APP susceptibility, the MIC was detected at 24, 36, 48, 72, 96, 120, 144, and 168 h by the agar-plate method. The ratio of bacterial MIC before and every 24 h after drug administration (MIC_final_/MIC_initial_) was calculated, and MIC_final_/MIC_initial_–time curves were created.

For measurement of the drug concentration, equal volumes of sample and acetonitrile were mixed and centrifuged (12,000 × *g*, 10 min, 4°C). Then, the supernatant (200 μL) was added to the mobile phase (800 μL), vortex-mixed, and passed through a 0.22-μm filter membrane for analysis by HPLC-MS/MS according to a protocol described previously (17). The limit of detection and limit of quantification were 5 and 10 ng/mL, respectively.

### 2.5 PK/PD analysis

The PK parameters AUC_168 h_ (area under the curve between 0 h to 168 h) and C_max_ (maximum concentration) were analyzed using a non-compartment model in WinNonlin.^1^

The antibacterial effect (I) was calculated as the maximum change in population (Log_10_ CFU/mL) over 168 h.

The PK/PD parameters AUC_168 h_/MIC_99_ and C_max_/MIC_99_ were calculated as the PK value divided by the MIC_99_. Also, %T > MIC_99_ (percentage of time that the tulathromycin concentration was above the MIC_99_ during 168 h) was obtained by PD-model analysis in WinNonlin.

The relationship between PK/PD parameters and I was analyzed using the inhibitory sigmoid E_max_ mode. The correlation coefficient (R^2^) was applied to represent the degree of fitting relationship (greater values reflect better correlation) according to the following formula:

$$I=I_{\text{max}}-\frac{\left(I_{\text{max}}-I_{o}\right)\times C_{e}^{N}}{C_{e}^{N}+IC_{50}^{N}}$$

where I represents the antibacterial effect as the change in bacterial count in different concentrations of drugs within 168 h (Log_10_ CFU/mL); I_max_ is the maximum antibacterial effect; I_0_ is the bacterial change in the blank group; C_e_ represents the PK/PD parameters AUC_168 h_/MIC_99_, C_max_/MIC_99_, and %T > MIC_99_; IC_50_ is the value of PK/PD parameters required to reach half of I_max_; N is the Hill coefficient, which represents the slope of the curve fitting PK/PD parameter and I.

According to the values of PK/PD parameters obtained, the PK/PD parameter values required to achieve bacteriostatic (0 Log_10_ CFU/mL reduction), bactericidal (3 Log_10_ CFU/mL), and elimination (4 Log_10_ CFU/mL) effects were calculated.

## 3 Results

### 3.1 MIC, MIC_99_, MPC_pr_, and MPC

The values for MIC, MIC_99_, MPC_pr_, and MPC of tulathromycin against APP were 2, 1.4, 64, and 44.8 μg/mL, respectively. The range of the MSW was 1.4–44.8 μg/mL. The selection index (MPC/MIC_99_) was 32.

### 3.2 PK of tulathromycin in the PPIM

**Figure 1:**
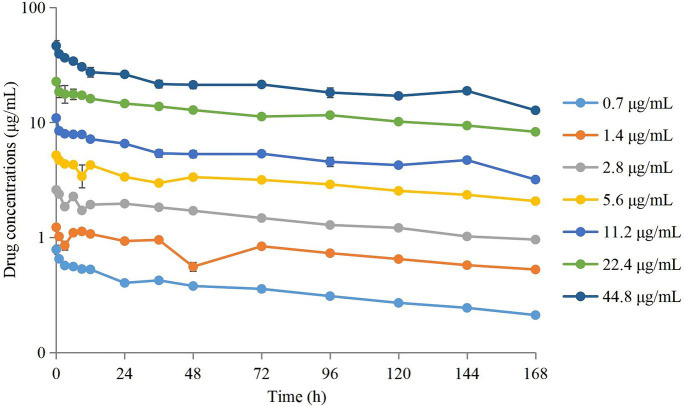
Concentration–times curves of tulathromycin in peristaltic pump infection model based on the PK in pig lungs. Values are the mean ± standard deviations (SD), *n* = 3.

The semi-logarithmic concentration–time curves of tulathromycin in the PPIM are shown in Figure 1. The main PK parameters after non-compartment analysis are shown in Table 1. C_max_ and AUC_0–168 h_ ranged from 0.78 ± 0.08 to 46.37 ± 5.08 μg/mL and from 57.68 ± 2.05 to 3489.91 ± 64.95 μg/mL, respectively, and R^2^ values between corresponding drug concentrations were 0.9997 and 0.995, respectively. The elimination half-life (T_1/2β_) ranged from 133.93 ± 5.59 h to 158.74 ± 8.68 h, with an average of 147.82 h, which was not significantly different from the set value (142 h).

**Table 1:** PK parameters of tulathromycin in our peristaltic pump infection model.

	Dosage groups (μg/mL)						
PK parameter (units)	0.7	1.4	2.8	5.6	11.2	22.4	44.8
_T1/2β_ (h)	133.93 ± 5.59	155.86 ± 18.31	136.87 ± 7.29	157.32 ± 3.27	145.33 ± 5.58	158.74 ± 8.68	146.67 ± 17.92
C_max_ (μg/mL)	0.78 ± 0.08	1.23 ± 0.07	2.60 ± 0.11	5.19 ± 0.01	10.93 ± 0.06	22.67 ± 0.24	46.37 ± 5.08
AUC_168h_ (μg•h/mL)	57.68 ± 2.05	125.01 ± 5.51	243.64 ± 4.82	499.60 ± 5.20	868.48 ± 15.78	1995.88 ± 27.20	3489.91 ± 64.95
AUC_infinity_ (μg•h/mL)	98.88 ± 2.33	243.24 ± 6.75	433.06 ± 16.88	969.82 ± 3.29	1536.73 ± 46.13	3894.53 ± 136.38	6188.38 ± 392.37
MRT_last_ (h)	70.86 ± 0.20	74.94 ± 0.40	72.13 ± 0.65	75.04 ± 0.63	74.30 ± 0.61	74.23 ± 0.80	73.94 ± 0.32

T_1/2β_, elimination half-life; C_max_, maximum concentration; AUC_168h_, AUC computed from time zero to 168 h; AUC_infinity_, AUC from time zero extrapolated to infinity; MRT_last_, mean residence time when the drug concentration is based on values up to and including the last measured concentration. Values are the mean ± standard deviations (SD), *n* = 3.

### 3.3 Time–kill curves of tulathromycin against APP

**Figure 2:**
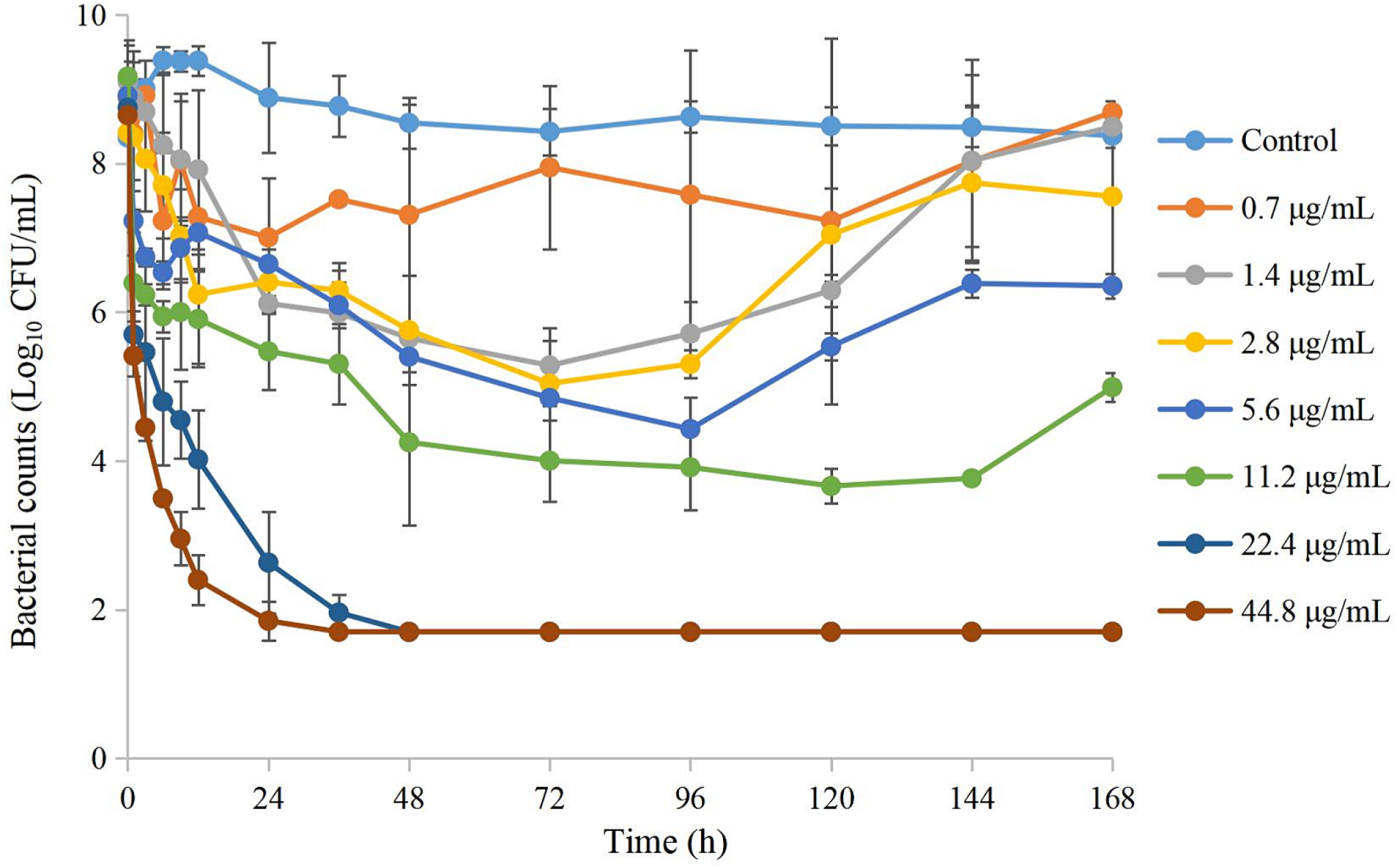
Time–kill curves of different concentrations of tulathromycin against *A. pleuropneumoniae* in a peristaltic pump infection model. Values are the mean ± standard deviations (SD), *n* = 3.

Time–kill curves of tulathromycin at different concentrations against APP are shown in Figure 2. The APP population decreased under treatment with different concentrations of tulathromycin, but recovered growth gradually at 1.4 μg/mL and 11.2 μg/mL. The antibacterial effects are shown in Table 2. A bacteriostatic effect was observed at 0.7 μg/mL (−1.88 Log_10_ CFU/mL). Bactericidal effects were achieved at 1.4 and 2.8 μg/mL (−3.82 and − 3.37 Log_10_ CFU/mL, respectively). An elimination effect without regrowth was observed at 5.6 to 44.8 μg/mL (−4.48 to −7.05 Log_10_ CFU/mL).

**Table 2:** Antibacterial effect (I) and PK/PD parameters of tulathromycin against APP.

Groups (μg/mL)	C_max_/MIC_99_	AUC_168 h_/MIC_99_ (h)	%T > MIC_99_ (%)	I (Log_10_ CFU/mL)
Control	0.00	0.00	0.00	1.04
0.7	0.56	41.20	0.00	−1.88
1.4	0.88	89.29	0.00	−3.82
2.8	1.85	174.03	48.57	−3.37
5.6	3.70	356.86	100.00	−4.48
11.2	7.81	620.34	100.00	−5.51
22.4	16.19	1425.63	100.00	−7.05
44.8	33.12	2492.79	100.00	−6.95

AUC_0–168 h_/MIC_99_, area under the curve between 0 h to 168 h divided MIC_99_; C_max_/MIC_99_, maximum concentration divided MIC_99_; %T > MIC_99_, percentage of time that the tulathromycin concentration was above the MIC_99_ during 168 h. All values were calculated as the mean value of three values.

### 3.4 Changes in APP susceptibility

**Figure 3:**
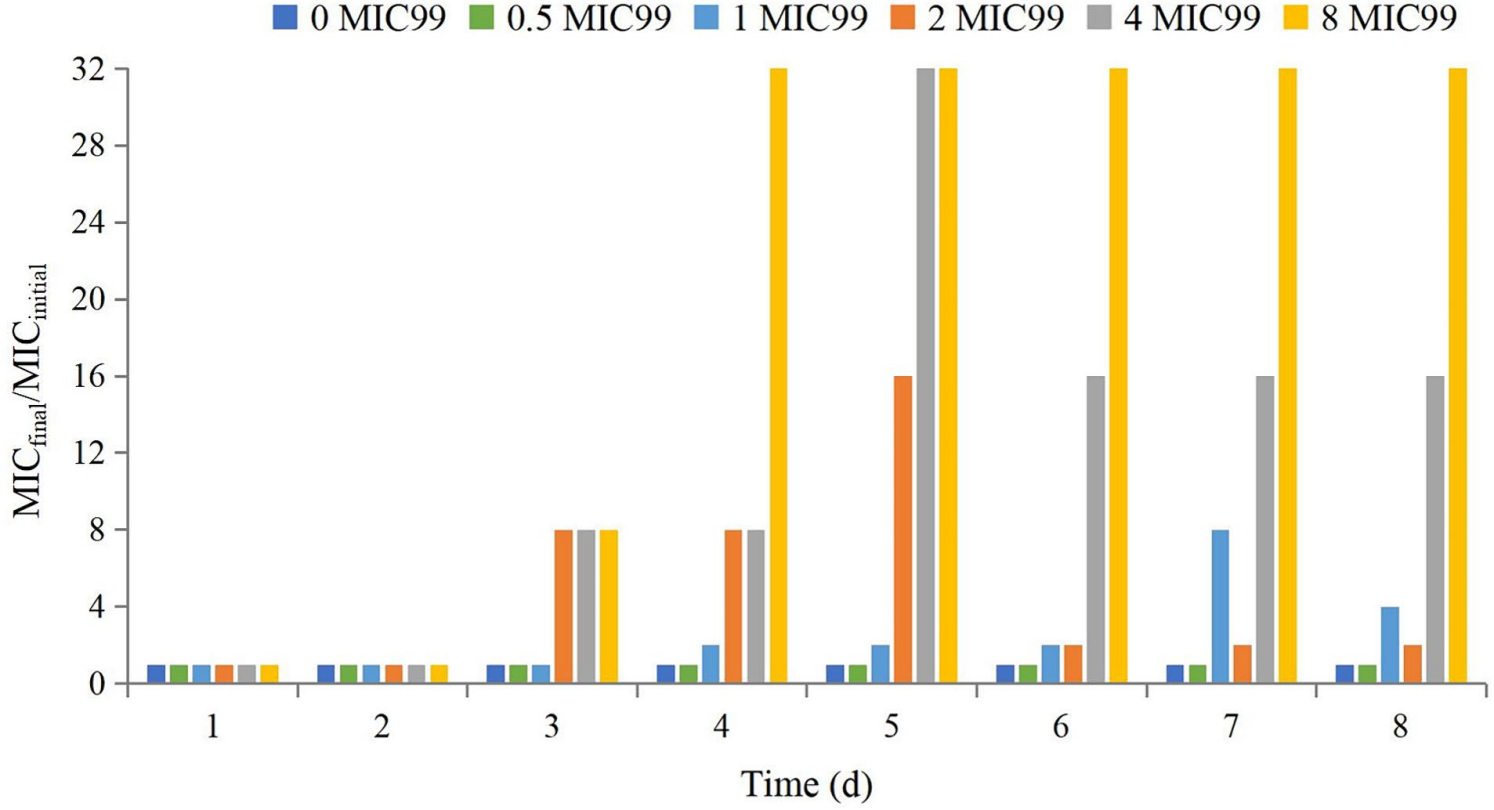
Values of MIC_final_/MIC_initial_ after drug administration at different times. MIC_final_ values represented the highest value after experiments were done in triplicate.

The MIC_final_/MIC_initial_ at different time points is shown in Figure 3. Seventy-two hours after drug administration, the MIC of APP increased 4-fold at 1 MIC_99_, 2-fold at 2 MIC_99_, 16-fold at 4 MIC_99_, and 32-fold at 8 MIC_99_. However, with the extension of time and decreasing drug concentrations, the MIC decreased to varying degrees except at 8 MIC_99_.

### 3.5 PK/PD integration

**Figure 4:**
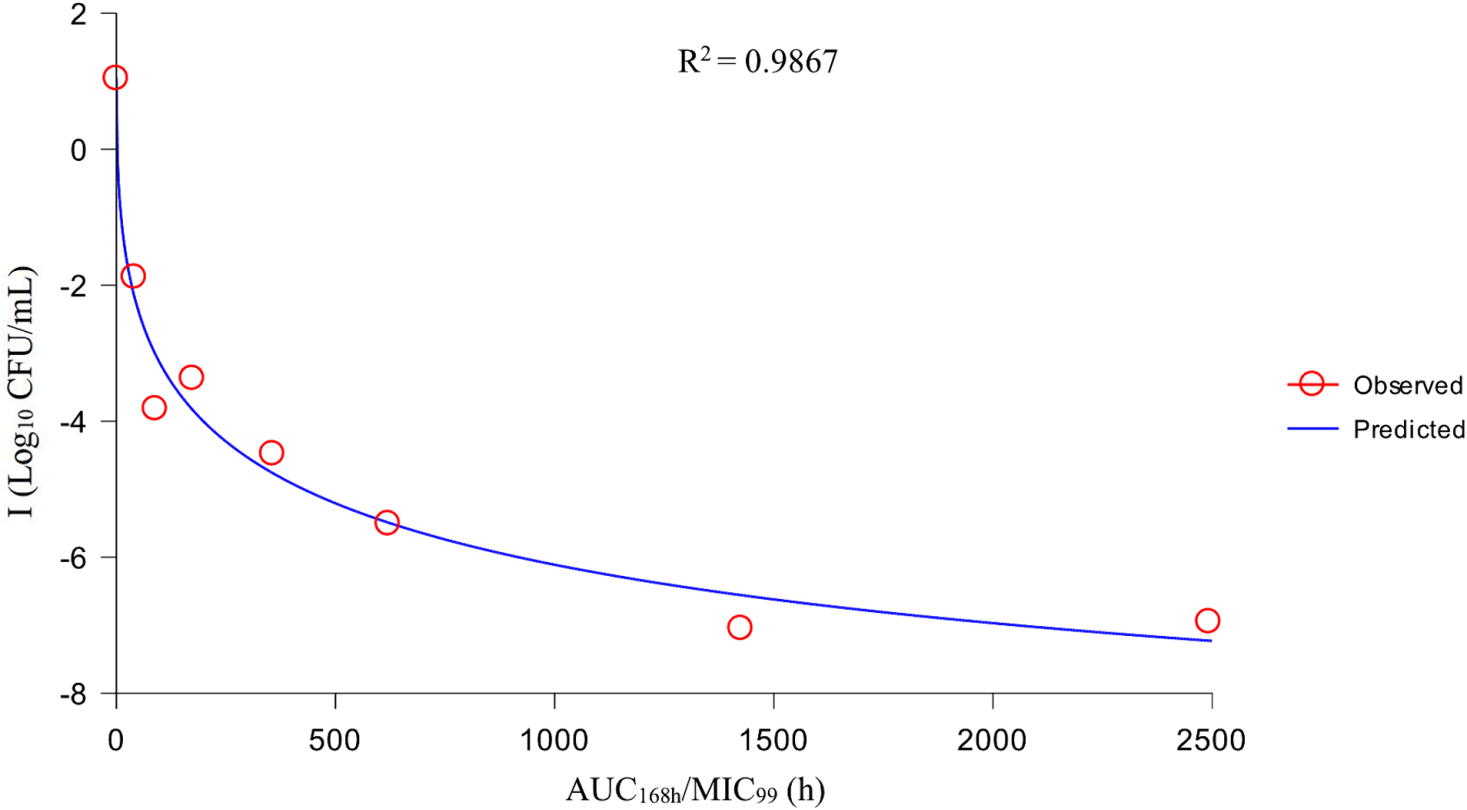
Fitting curve between AUC_168 h_/MIC_99_ and antibacterial effect (I).

**Figure 5:**
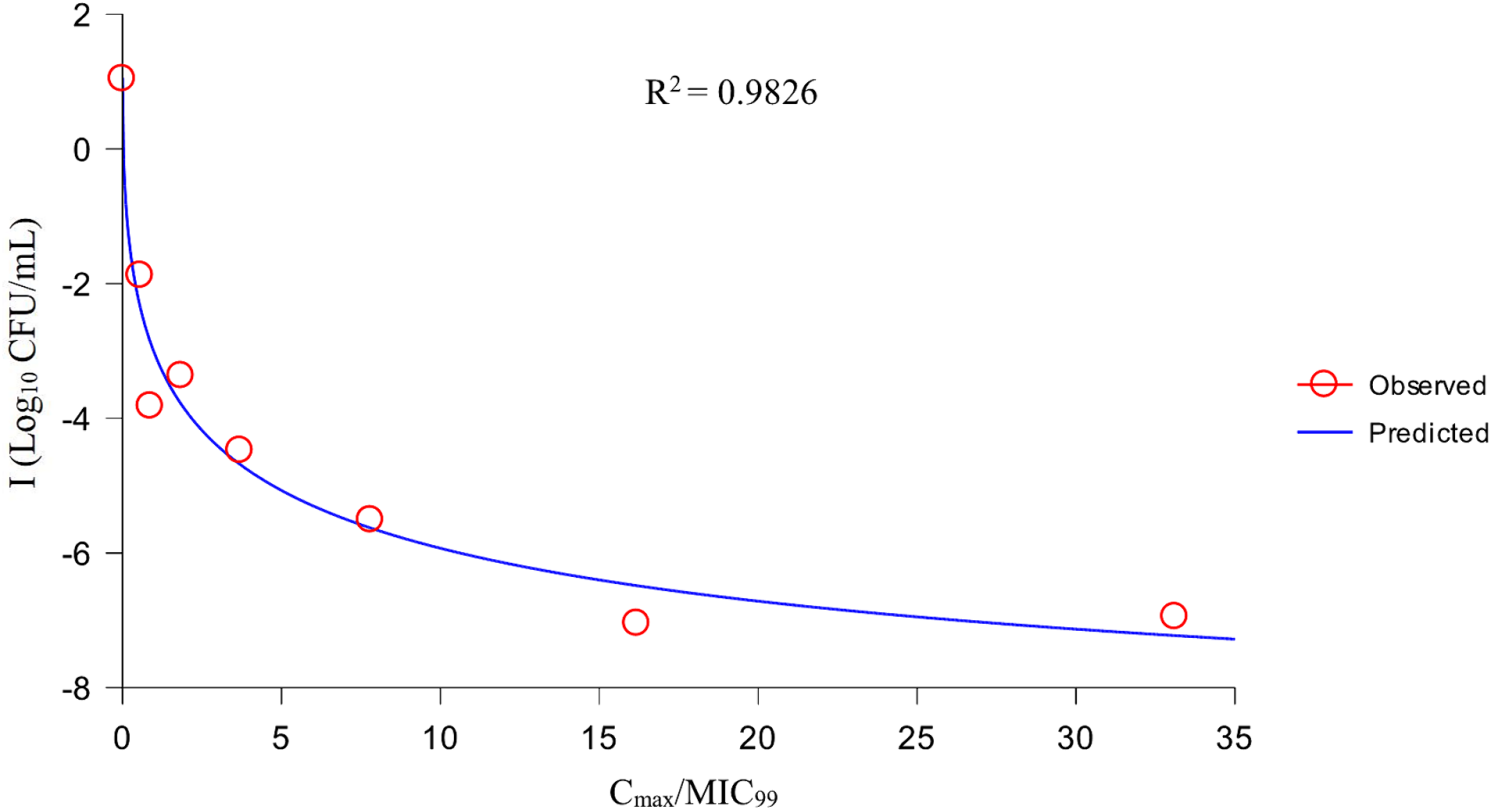
Fitting curve between C_max_/MIC_99_ and antibacterial effect (I).

After analysis the relationships between PK/PD parameters and antibacterial effects, AUC_168 h_/MIC_99_ showed the best fit to I (*R*^2^ = 0.9867) compared with C_max_/MIC_99_ (0.9826) and %T > MIC_99_ (0.8168), and the fitting curves of AUC_168 h_/MIC_99_ C_max_/MIC_99_ are shown in Figures 4, 5, respectively. The fitted PK/PD parameters and predicted values of AUC_168 h_/MIC_99_ for achieving different antibacterial effects are shown in Table 3. The AUC_168 h_/MIC_99_ required to achieve bacteriostatic, bactericidal, and eradication effects was 1.80, 87.42, and 198.00 h, respectively.

**Table 3:** PK/PD parameters and AUC_168 h_/MIC_99_ to achieve different antibacterial effects.

PK/PD parameters	Values
I_max_ (Log_10_ CFU/mL)	1.04
IC_50_ (h)	450.92
I_max_−I_0_ (Log_10_ CFU/mL)	−12.22
Slope (N)	0.43
AUC_168 h_/MIC_99_ for bacteriostatic effect (h)	1.80
AUC_168 h_/MIC_99_ for bactericidal effect (h)	87.42
AUC_168 h_/MIC_99_ for eradication effect (h)	198.00

I_max_, maximum antibacterial effect; I_0_, bacterial change in blank group; IC_50_, value of AUC_0-168 h_/MIC_99_ required to reach half of I_max_; N, Hill coefficient, which represents the slope of the curve fitting AUC_0–168 h_/MIC_99_ and I.

## 4 Discussion

The gradual large-scale and intensive development of the farming industry have led to disease outbreaks. Hence, many types of antimicrobial drugs are used to prevent and treat infections in animals.

However, drug-resistant bacteria have emerged and spread worldwide, which seriously threatens the life and health of humans and animals. One report which studied the antimicrobial-resistance profiles of APP in the UK revealed that 57% of bacteria were resistant to tetracycline, 48% to sulfisoxazole, 20% to ampicillin, 17% to trimethoprim, and 6% to enrofloxacin (20). In the face of the threat of drug-resistant bacteria, the most common approach is to develop new veterinary drugs and optimize drug-delivery schedules. However, the development speed of new veterinary drugs cannot keep pace with the mutation speed of drug-resistant bacteria. Therefore, optimizing drug-delivery schemes is the most practical and reliable way to deal with the threat of drug-resistant bacteria. The PK/PD synchronous model is used to analyze relationships between host, pathogen, and drugs. It has been applied widely for optimization of dosage regimens (9, 11, 12).

The PK/PD of tulathromycin against various pathogenic bacteria has been studied. Zhou et al. (21) applied a tissue-cage model in pigs to investigate the PK/PD integration of tulathromycin against *Pasteurella multocida*. They found that AUC_(0–24 h)_/MIC was the fittest PK/PD parameter for predicting the antibacterial effect (*R*^2^ = 0.9969) and the values required to achieve bacteriostatic, bactericidal, and eradication effect were 44.55, 73.19, and 92.44 h in serum, 23.17 h for a bacteriostatic effect in transudates, and 32.42, and 41.85 h for bacteriostatic and bactericidal effects in exudates, respectively. Guo et al. (22) researched the PK/PD of tulathromycin against *Haemophilus parasuis* in a lung-infection model in guinea pigs. They showed that the AUC_168 h_/MIC required to achieve a reduction of 1, 2, 3, and 4 Log_10_ CFU/mL of *H. parasuis* was 366.06, 552.51, 728.47, and 916.90 h in serum, and 507.20, 1,227.11, 2,126.44, and 3,462.62 h in lung tissue, respectively. Yao et al. (17) studied the PK/PD of tulathromycin against APP in a tissue-cage infection model. They discovered that %T > MIC was the best PK/PD parameter for predicting the antibacterial effect (*R*^2^ = 0.9421) and the values required to achieve a reduction of 1 Log_10_ CFU/mL and 3 Log_10_ CFU/mL were 50.8 and 96.38%, respectively. However, that study could not reflect the actual antibacterial effect in clinical infection because the PK of a drug is different between tissue fluid and the lung (target tissue in APP infection). Obtaining real-time and continuous PK and PD in pig lungs is difficult. The PPIM can simulate the dynamic changes in the drug concentration based on *in vivo* PK, which can obtain a real-time antibacterial effect, especially for simulating the PK and PD in difficult-to-obtain organs.

Therefore, we employed a PPIM to carry out a MSW-based PK/PD study of tulathromycin against APP by simulating the PK of tulathromycin in pig lungs. The C_max_ and AUC_168 h_ obtained showed a linear correlation with the corresponding drug concentrations, with *R*^2^ values of 0.9997 and 0.995, respectively. The mean realistic T_1/2β_ value (147.82 h) determined in our study was no significant difference to the initial set value (142 h). These results showed that our PPIM was established and could operate stably. After PK/PD analysis, AUC_168 h_/MIC_99_ showed the best fit to the antibacterial effect, with *R*^2^ = 0.9867 (C_max_/MIC_99_, *R*^2^ = 0.9826; %T > MIC_99_, *R*^2^ = 0.8168). The AUC_168 h_/MIC_99_ required to achieve bacteriostatic, bactericidal, and clearance effects was 1.80, 87.42, and 198.00 h, respectively. Thus, tulathromycin exhibited time-dependent and concentration-dependent activities against APP.

In MSW theory, if the drug concentration is between the MIC and MPC, then sensitive bacteria are killed and insensitive bacteria multiply in large numbers and may produce gene mutations induced by the subinhibitory concentration, which may result in serious drug resistance. The MSW is an important guiding principle of a drug-administration regimen, and has been studied extensively. Lozano-Huntelman et al. (23) analyzed the evolution of the MIC and MPC of seven drugs against *Staphylococcus epidermidis* and its mutants. They found that the MSW of mutant bacteria shifted to the right and increased breadth, which is an important guide for predicting the evolutionary trajectory of drug-resistant bacteria. Shi et al. (24) studied the MSW of ciprofloxacin against *Pseudomonas aeruginosa*. If drug concentrations were located in the middle of the MSW, the bacterial genes *rhlR* and *pqsR* were mutated and the locomotion ability and biofilm-secretion ability of the mutant bacteria were enhanced significantly. Golikova et al. (25) predicted the relationship between the MPC of daptomycin and rifampicin against *S. aureus* and drug-resistant bacteria using an MPC-based parameter: area under the drug–time curve above and below the MPC. They predicted the concentration region to prevent the enrichment of mutant bacteria.

We found that the sensitivity of APP was reduced significantly if the drug concentration was in the middle or lower part of the MSW. The final MIC was increased 2–32-fold compared with the initial value, with corresponding AUC_168 h_/MIC_99_ of 89.29 and 620.34 h, respectively. Especially when the value of AUC_168h_/MIC_99_ were located between 356.86 and 620.34 h, the MIC was increased above 16 fold. These results are similar to data from other studies (26–28). There are two reasons for this phenomenon. First, in the original flora, sensitive bacteria are the dominant bacteria, but there are some insensitive drug-resistant bacterial subpopulations. If the drug concentration is between MIC_99_ and MPC, sensitive bacteria are killed gradually after multiple administrations and the number of insensitive bacteria increases gradually and becomes the dominant flora. Second, sensitive bacteria and insensitive bacteria had gene mutations under the continuous pressure of drug selection. APP may have multiple drug-resistant gene mutations, which could make it more resistant to tulathromycin. Therefore, if designing a dosage regimen, the AUC_168 h_/MIC_99_ produced should not be between 356.86 h and 620.34 h.

## 5 Conclusion

A PPIM was applied to study the MSW of tulathromycin against APP. We showed that this model ran stably and could simulate the PK characteristics of tulathromycin in pig lungs. AUC_168 h_/MIC_99_ had the best fit with the antibacterial effect (*R*^2^ = 0.9867). The AUC_168 h_/MIC_99_ required to achieve bacteriostatic, bactericidal, and clearance effects was 1.80, 87.42, and 198.00 h, respectively. However, if the drug concentration was in the middle or lower part of the MSW, then bacterial sensitivity was reduced significantly. Therefore, if administering tulathromycin, the concentration needed to induce bacterial mutations should be avoided. To achieve a therapeutic effect, the dose should be adjusted to produce AUC_168 h_/MIC_99_ < 356.86 h or > 620.34 h. These findings could guide the clinical application of tulathromycin in the treatment of APP infection to avoid the generation and amplification of drug-resistant bacteria.

## Data availability statement

The raw data supporting the conclusions of this article will be made available by the authors, without undue reservation.

## Author contributions

HW: Data curation, Formal analysis, Investigation, Methodology, Project administration, Writing – original draft. LZ: Data curation, Formal analysis, Software, Supervision, Writing – review & editing.

## Footnotes

^1^ www.certara.com/software/phoenix-winnonlin/
